# Involvement of the Rostromedial Prefrontal Cortex in Human-Robot Interaction: fNIRS Evidence From a Robot-Assisted Motor Task

**DOI:** 10.3389/fnbot.2022.795079

**Published:** 2022-03-17

**Authors:** Duc Trung Le, Kazuki Watanabe, Hiroki Ogawa, Kojiro Matsushita, Naoki Imada, Shingo Taki, Yuji Iwamoto, Takeshi Imura, Hayato Araki, Osamu Araki, Taketoshi Ono, Hisao Nishijo, Naoto Fujita, Susumu Urakawa

**Affiliations:** ^1^Department of Musculoskeletal Functional Research and Regeneration, Graduate School of Biomedical and Health Sciences, Hiroshima University, Hiroshima, Japan; ^2^Department of Neurology, Vietnam Military Medical University, Hanoi, Vietnam; ^3^Department of Mechanical Engineering, Facility of Engineering, Gifu University, Gifu, Japan; ^4^Department of Rehabilitation, Araki Neurosurgical Hospital, Hiroshima, Japan; ^5^Department of Rehabilitation, Faculty of Health Sciences, Hiroshima Cosmopolitan University, Hiroshima, Japan; ^6^Department of Neurosurgery, Araki Neurosurgical Hospital, Hiroshima, Japan; ^7^Department of System Emotional Science, Faculty of Medicine, University of Toyama, Toyama, Japan; ^8^Research Center for Idling Brain Science (RCIBS), University of Toyama, Toyama, Japan

**Keywords:** frontal pole, prefrontal cortex (PFC), NIRS (near-infrared spectroscopy), Hybrid Assistive Limb (HAL®), assistive exoskeleton robot, executive function

## Abstract

Assistive exoskeleton robots are being widely applied in neurorehabilitation to improve upper-limb motor and somatosensory functions. During robot-assisted exercises, the central nervous system appears to highly attend to external information-processing (IP) to efficiently interact with robotic assistance. However, the neural mechanisms underlying this process remain unclear. The rostromedial prefrontal cortex (rmPFC) may be the core of the executive resource allocation that generates biases in the allocation of processing resources toward an external IP according to current behavioral demands. Here, we used functional near-infrared spectroscopy to investigate the cortical activation associated with executive resource allocation during a robot-assisted motor task. During data acquisition, participants performed a right-arm motor task using elbow flexion-extension movements in three different loading conditions: robotic assistive loading (ROB), resistive loading (RES), and non-loading (NON). Participants were asked to strive for kinematic consistency in their movements. A one-way repeated measures analysis of variance and general linear model-based methods were employed to examine task-related activity. We demonstrated that hemodynamic responses in the ventral and dorsal rmPFC were higher during ROB than during NON. Moreover, greater hemodynamic responses in the ventral rmPFC were observed during ROB than during RES. Increased activation in ventral and dorsal rmPFC subregions may be involved in the executive resource allocation that prioritizes external IP during human-robot interactions. In conclusion, these findings provide novel insights regarding the involvement of executive control during a robot-assisted motor task.

## Introduction

Stroke represents the second most common cause of mortality and the third most common cause of disability worldwide (Lozano et al., 2012). Despite considerable progress in the management of acute stroke, many stroke survivors experience various functional deficits, which severely affect their ability to fulfill daily tasks. Robotic devices have emerged as rehabilitation tools capable of providing task-specific, intensive, and multi-sensory training (Sivan et al., 2011; Turner et al., 2013; Calabrò et al., 2016) while reducing manpower and labor requirements during the therapeutic process. Among the multiple types of robots developed, assistive exoskeleton robots based on human motion intention detection represent a promising technology, given their ability to augment the performance and improve the motor function of users (Godfrey et al., 2013; Klamroth-Marganska et al., 2014; Sale et al., 2014; Takahashi et al., 2016). Due to their direct reflection of a human movement intention, bioelectrical signals from muscle regions have been commonly employed in techniques used for controlling assistive exoskeleton robots (e.g., Hybrid Assistive Limb system) (Sankai and Sakurai, 2018). Additionally, exoskeleton robots can also be controlled using a performance-based method, in which assistive forces are adjusted to support the user's movement based on his or her motor performance (e.g., InMotion 2.0 system) (Krebs et al., 2003). Brain-controlled exoskeletons have been used as brain-computer interfaces (BCIs) in assistive exoskeleton robots to decode brain processes from brain signals, such as electroencephalography (EEG) (Hong and Khan, 2017; Choi et al., 2020) or hemodynamic signals (Khan and Hong, 2017; Asgher et al., 2021), and convert them into output motor commands (e.g., BCI-Manus system). Determining the effects of robot-assisted rehabilitation requires a deeper understanding of the mechanisms underlying human-robot interactions.

Since processing resources are limited (Miller and Cohen, 2001), the executive processes that direct the central nervous system to the most relevant information processing (IP) according to the current behavior constitute an important feature of high-level function (Burgess et al., 2007a; Dixon et al., 2014). Regarding the motor domain, external IP deals with behavior that requires interaction with external influences, while internal IP occurs during self-generated or -paced movements. A series of earlier studies by Burgess et al. proposed that when the human mind is occupied with a given task, it involves high-level processes that govern the allocation of processing resources (Burgess et al., 2007a,b; Burgess and Wu, 2013). In particular, such processes engage an executive control system centered on the rostral prefrontal cortex (also known as the frontopolar cortex) to evaluate the ongoing behavior and bias the allocation of processing resources toward the IP relevant to the current behavioral goal, termed as executive resource allocation. Under this framework, the lateral part of the rostral PFC (rlPFC) biases toward internal IP, whereas the medial part of the rostral PFC (rmPFC), especially its most anterior subdivision, prioritizes external IP. Supporting this hypothesis, converging neuroimaging evidence has revealed increased activation in the rmPFC and rlPFC during task performance influenced by external and internal IP, respectively (Gilbert et al., 2006, 2007; Simons et al., 2008; Henseler et al., 2011).

Multiple lines of evidence suggest that performing a motor task with an assistive exoskeleton robot requires the central nervous system to focus on external IP. For instance, assistive exoskeleton robots provide physical support for the subject to perform a task *via* assessing their motion intentions in real-time (Lenzi et al., 2012). Therefore, the somatosensory response generated by the external physical coordination of the human limb with the assistive exoskeleton robot plays an important role in the adaptive control of human-robot interaction. Furthermore, robot-assisted motor learning is strongly associated with augmented somatosensory feedback processing (Sigrist et al., 2013; Maeda et al., 2018). Human-robot interactions specifically yield robust somatosensory feedback of assisted movements to the central nervous system, facilitating improved generation of internal dynamic models for movement guidance in the performer (Sigrist et al., 2013). These processes form closed-loop action-perception pathways that promote sensorimotor stimulation, which drives brain plasticity. Based on the above findings, the processing of external information contributes substantially to motor performance and learning by assistive exoskeleton robots. This necessitates prioritizing external IP during human-robot interactions, thereby involving the executive resource allocation that depends on the rmPFC. Although sensorimotor system involvement in robot-assisted motor tasks has been investigated extensively (Kim et al., 2016; Saita et al., 2017, 2018; Simis et al., 2018; Berger et al., 2019), the underlying high-level control mechanisms that govern the allocation of processing resources during a motor task with an assistive exoskeleton robot have not been elucidated.

The role of the rmPFC in top-down executive control in the performance of novel motor tasks has been investigated in a variety of study contexts. For example, several functional near-infrared spectroscopy (fNIRS) studies have reported that the involvement of high-level cognitive activities of PFC areas, including the rmPFC, is involved in learning novel upper limb motor skills (Ishikuro et al., 2014; Kobayashi et al., 2021). Recent work on fNIRS-based neurofeedback training also found that the anterior PFC, which corresponds to the rmPFC in this study, plays a role in top-down modulation of activity in the sensorimotor cortex in order to optimize motor performance (Ota et al., 2020). Studies on BCI have also shed light on rmPFC functions. In fNIRS-based BCI systems, rmPFC hemodynamic signals during motor imagery and execution have been used to detect user's intention of movements (Naseer and Hong, 2015; Hong et al., 2017; Peng et al., 2018a; Khan et al., 2020). Movement-related cortical potentials have also been successfully decoded from EEG-based signals in the rmPFC (Min et al., 2017; Koizumi et al., 2018). These signals are useful in controlling brain-controlled exoskeletons designed to augment the user's sensorimotor functions (Agashe et al., 2016; Hong and Khan, 2017; Khan and Hong, 2017; Liu et al., 2018; Asgher et al., 2021). Moreover, a limited number of studies have been conducted to investigate the effect of robot-assisted tasks on cortical reorganization (Youssofzadeh et al., 2016; Saita et al., 2017, 2018; Memar and Esfahani, 2018; Berger et al., 2019; Peters et al., 2020). Most of these investigations, however, did not look at the rmPFC activity. Notably, A few EEG studies have reported significant functional connectivity among brain regions within the fronto-centro-parietal network during robot-assisted gait training (Youssofzadeh et al., 2016; Memar and Esfahani, 2018). This network has been regarded as the top-down executive control network, with the rmPFC area being a higher-order component (Peng et al., 2018b), may be involved in human-robot interactions. Despite the findings of the previous studies, the role of the rmPFC in top-down executive control over human-robot interactions remains unclear.

fNIRS is emerging as a practical imaging tool for assessing the cortical activity of the cerebrum during motion-demanding tasks. fNIRS is a non-invasive and low-cost optical technique that measures concentration changes in oxygenated hemoglobin (oxy-Hb) and deoxygenated hemoglobin (deoxy-Hb) in cortical microvasculature. fNIRS signals have been proven to be associated with cortical activation (Okamoto et al., 2004; Hoge et al., 2005) and BOLD signals (Cui et al., 2011; Scarapicchia et al., 2017), indicating the feasibility of fNIRS analysis to detect human cortical activity. Because this modality has no restriction on the participant and high tolerance against motion artifacts, fNIRS-based experiments are carried out in unconstrained settings with free limb movements, allowing for more robust reproductions of genuine cognitive processes. fNIRS, therefore, has been widely applied in many motor control studies involving upper limb movement (Ishikuro et al., 2014), walking (Mihara et al., 2007), and social interaction (Urakawa et al., 2015). Thus, fNIRS is an optimal neuroimaging approach for investigating the cortical activity of the cerebrum during human-robot interactions.

The present study aimed to investigate the cortical activity associated with executive resource allocation during a motor task with an assistive exoskeleton robot. We employed a single-joint version of the Hybrid Assistive Limb (HAL-SJ) system, a wearable assistive exoskeleton robot developed to aid upper and lower limb functions (Sankai and Sakurai, 2018). HAL-SJ provides robotic assistance for human limb movement by detecting bioelectrical signals from extensor-flexor muscle regions (Morishita and Inoue, 2016; Suzuki et al., 2016). Elbow flexion-extension movements that require kinematic consistency were adopted as the primary task. During fNIRS recordings, healthy participants prepared for and performed the motor task in three loading conditions requiring different degrees of external IP in ascending order: non-loading (NON), resistive loading (RES), and robotic assistive loading (ROB) condition. Investigating brain activation during robot-assisted motor tasks in healthy participants will aid in understanding how this task activates the brain differently across clinical groups. The outcomes of this study would serve as a baseline for future research to further investigate the neural mechanisms of human-robot interactions in healthy and disease-affected populations. We hypothesized that by providing robotic assistance to voluntary elbow movements, the executive process would be involved in generating bias for the allocation of processing resources toward external IP during the motor task, resulting in greater activation of rmPFC subregions during ROB compared to other loading conditions. To the best of our knowledge, this is the first study using fNIRS to assess the cortical manifestation of the executive resource allocation during a robot-assisted motor task.

## Materials and Methods

### Participants

In total, 26 healthy participants (13 females and 13 males, age range: 22.12 ± 1.34 years) were enrolled in this study. Participants were right-hand dominant as assessed using the Edinburg Handedness Questionnaire (88.74 ± 15.63). None of the participants reported a medical history of neurological or psychiatric disorders, or any orthopedic injuries that impaired upper limb sensorimotor function. Participants were instructed to avoid consuming any caffeine or alcohol-containing substances for at least 12 h prior to experiments. Participants were informed about the study's purpose and provided written informed consent before participation. All procedures were in compliance with the Declaration of Helsinki and the United States Code of Federal Regulations for the protection of human participants. The present study was approved by the Human Ethics Committee of Hiroshima University (No. C-114).

### Apparatus

**Figure 1:**
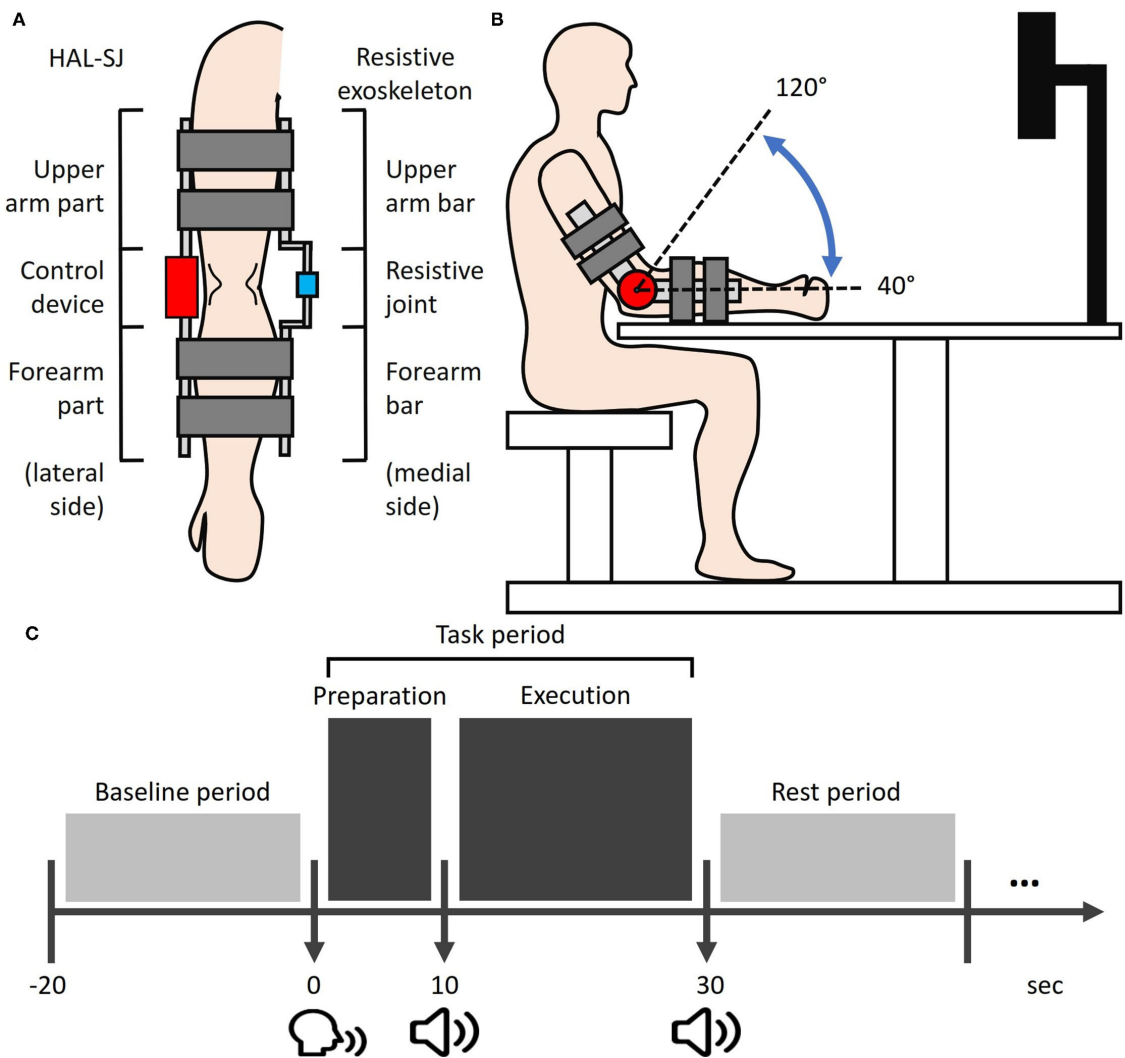
Experimental setup. **(A)** The HAL-SJ and the resistance exoskeleton device are mounted on the lateral and medial sides of the right arm, respectively. **(B)** Experimental setup from a sagittal view. During the task execution phase, participants were required to perform cyclic elbow flexion-extension movements with the ROM from 40 to 120° (dash line). **(C)** The simplified schematic of the experimental trial protocol. Each task period was 30 s long, including a 10-s preparation phase and a 20-s execution phase. The post-task period was pseudo-randomized between 20, 30, and 40 s. HAL-SJ, single-joint version of the Hybrid Assistive Limb; ROM, range-of-motion.

The HAL-SJ (Cyberdyne Inc., Tsukuba, Irabaki, Japan) is a wearable assistive exoskeleton robot that can be attached to the elbow or knee to support flexion and extension joint motions. The Cybernic Voluntary Control mode in HAL-SJ provides assistive forces to facilitate joint movements based on human motion intentions for human motion detected *via* bioelectrical signals from muscles. In this study, the HAL-SJ was set on the lateral side of each participant's right arm with two pairs of surface electrodes attached to the muscle belly of the biceps and triceps brachii muscles (Figure 1A). The setting parameters for HAL-SJ were standardized across participants, with an assistive gain of 45% and an assistive balance of zero between flexor and extensor motions.

A self-built resistance exoskeleton device was designed to apply constant resistive force on voluntary elbow movements of the right elbow. The resistance exoskeleton device was set on the medial side of the right arm and consisted of a 1°-of-freedom elbow joint with two bars attached to the arm and forearm (Figure 1A). During experiments, the resistance degree imposed by the device was controlled at ~95 Nm/rad for all participants.

### Task Conditions

Participants were seated in an upright position facing an LCD monitor (23 inches; Flexscan EV 2316V, Eizo, Japan) with a participant-monitor distance of 90 cm. Participants placed their right elbow on an elbow-rest and aligned the right arm to the starting posture, in which the arm was in line with the horizontal plane, with a 30°-flexed shoulder and a 40°-flexed elbow (Figure 1B). A chinrest was used to limit participants' head motion. As the present study focused on the information-processing of somatosensory feedback induced by human-robot interactions, participants were required to visually fixate on a cross displayed at the center of a monitor throughout the experiments in order to focus on somatosensory feedback while minimizing visual feedback during motor performance. Three different loading conditions for right elbow movements were assigned to each participant in a pseudo-randomized order: NON, RES, and ROB conditions. In the NON condition, elbow movements were self-controlled without external loading, demanding minimal operation of external IP. In the RES condition, a self-built resistance exoskeleton device was used to impose constant resistive force on elbow movements, apparently involving a higher level of external IP than NON. In the ROB condition, elbow movements were performed using a combined configuration of HAL-SJ and the resistance exoskeleton device (Figure 1A), which applied assistive and resistive forces on joint motions. The HAL-SJ was set to the Cybernic Voluntary Control mode which allows it to provide real-time external assistance to elbow movements by detecting human motion intention *via* bioelectrical signals from muscle activation. During ROB, the physical interaction between voluntary movement and intention-based robotic assistance may induce new and complex sensory feedback, requiring the highest degree of external IP among the three conditions.

### Task Procedure

Each session consisted of four consecutive trials in a block paradigm, carried out as follows: Baseline period—Task period—Rest period (Figure 1C). Each task trial commenced with a baseline period, during which participants were required to relax their right arm for 20 s while baseline hemodynamic responses were recorded. We designed a 30-s task period comprising two phases: a 10-s task preparation and a 20-s task execution. The start of the preparation phase was signaled by a verbal command “10 s left” from the experimenter, during which participants were instructed to prepare for the upcoming motor task without performing any actual movements or muscle contractions. The execution phase commenced after a beep was presented. Participants were then allocated 20 s to perform cyclic elbow flexion-extension movements with a range of motion (ROM) from ~40° to 120° (Figure 1B). Movement frequency was limited to 0.5–1 Hz. Participants were encouraged to strive for kinematic consistency in their ROM of flexion-extension movements, referred to as task performance. The task period ended after two beeps, which cued participants to return their arms to the starting posture and rest until the next trial.

A complete experimental session lasted ~4 min, with a 5-min inter-session interval for setting up the new condition. The total experimental duration was 25 min per participant. After completing the experimental conditions, participants were required to complete a VAS that examined subjective measures of effort on the task performance (from “very low degree” to “very high degree”) for each condition. Prior to participation, participants were familiarized with the task by undergoing several practice trials for each condition.

### Behavioral Data Acquisition and Exclusion Criteria

To measure the absolute angle information of the forearm, a motion sensor (MPU9250; TDK InvenSense, CA, USA) was set up at the right wrist. The device featured 16-bit data outputs in the range of ±2 G for each of the three acceleration axes and ±2,000 degrees per second (dps) for each of the three gyroscopic axes, with a sampling frequency of 500 Hz. A microcontroller (ARM mbed LPC1768; NXP Semiconductors, Eindhoven, Netherlands) input the acquired data to a Madgwick filter, calculated the attitude estimation of the absolute angle of the forearm with a sampling frequency of 20 Hz, and sent the absolute angle information to a PC. The ROM for each cyclic elbow movement was determined by subtracting the minimal angle from the maximum angle. The standard deviation (SD) of the ROM for each trial was generated across all movements. For statistical analysis, kinematic variability in each condition was quantified by calculating the average SD values across trials.

Behavioral performance was recorded using a video recording device (iPhone; Apple Inc., CA, USA). To additionally assess the quality of interaction between voluntary movements of participants and external assistance of the HAL-SJ, the number of jerks during ROB performance was measured for each trial using visual inspection. For statistical analysis, the mean number of jerks across four trials was calculated for each participant.

To confirm the participant's compliance with task instructions during the experiment, their behaviors were evaluated visually and electromyographically. To monitor the muscular activity of arm muscles, bipolar surface electrodes with an inter-electrode distance of 10 mm were placed over the muscle belly of the right bicep and tricep muscles. A reference electrode was attached to the left wrist. EMG signals were acquired with a band-pass filter from 10 to 500 Hz at a sampling rate of 1 kHz using an EMG system (EMG Master; Mediarea Support Business Union, Okayama, Japan). The processed EMG tracings were visually inspected to detect muscle contractions. One individual participant data with more than two trials containing incorrect behaviors such as poor performance, marked body movements, lack of attention, or noticeable muscle activity during non-execution periods in any condition were excluded from subsequent data processing and statistical analyses. As a result, there was a total of the remaining 18 participants analyzed (10 females and 8 males, age range: 22.11 ± 1.49 years).

### fNIRS Data Acquisition

A multi-channel fNIRS system (FOIRE-3000; Shimadzu, Kyoto, Japan) was employed to measure cortical hemodynamic activity during experiments with a sampling rate of 7.69 Hz or a temporal resolution of 130 ms. The temporal resolution used in this study is relatively comparable to that used in previous fNIRS studies of human-robot interactions (Saita et al., 2018; Berger et al., 2019). Given that task-related brain activities were assessed across a 10-s interval or longer, a sampling rate of 7.69 Hz offered adequate bandwidth to detect changes in hemodynamic responses. Specifically, the measured changes in light absorption recorded at three wavelengths (780, 805, and 830 nm) *via* semiconductor laser diodes were transformed into corresponding concentration changes in oxy-Hb, deoxy-Hb, and total hemoglobin (total-Hb) using the modified Beer–Lamberts law (Delpy et al., 1988). These values were assessed using the unit of molar concentration multiplied by length (mM × mm). Given that changes in oxy-Hb signal are the most sensitive indicator of changes in regional cortical blood flow and have the highest signal-to-noise ratio (Okamoto et al., 2004; Hoge et al., 2005), the analysis and discussion in this study focused primarily on changes in oxy-Hb concentration.

**Figure 2:**
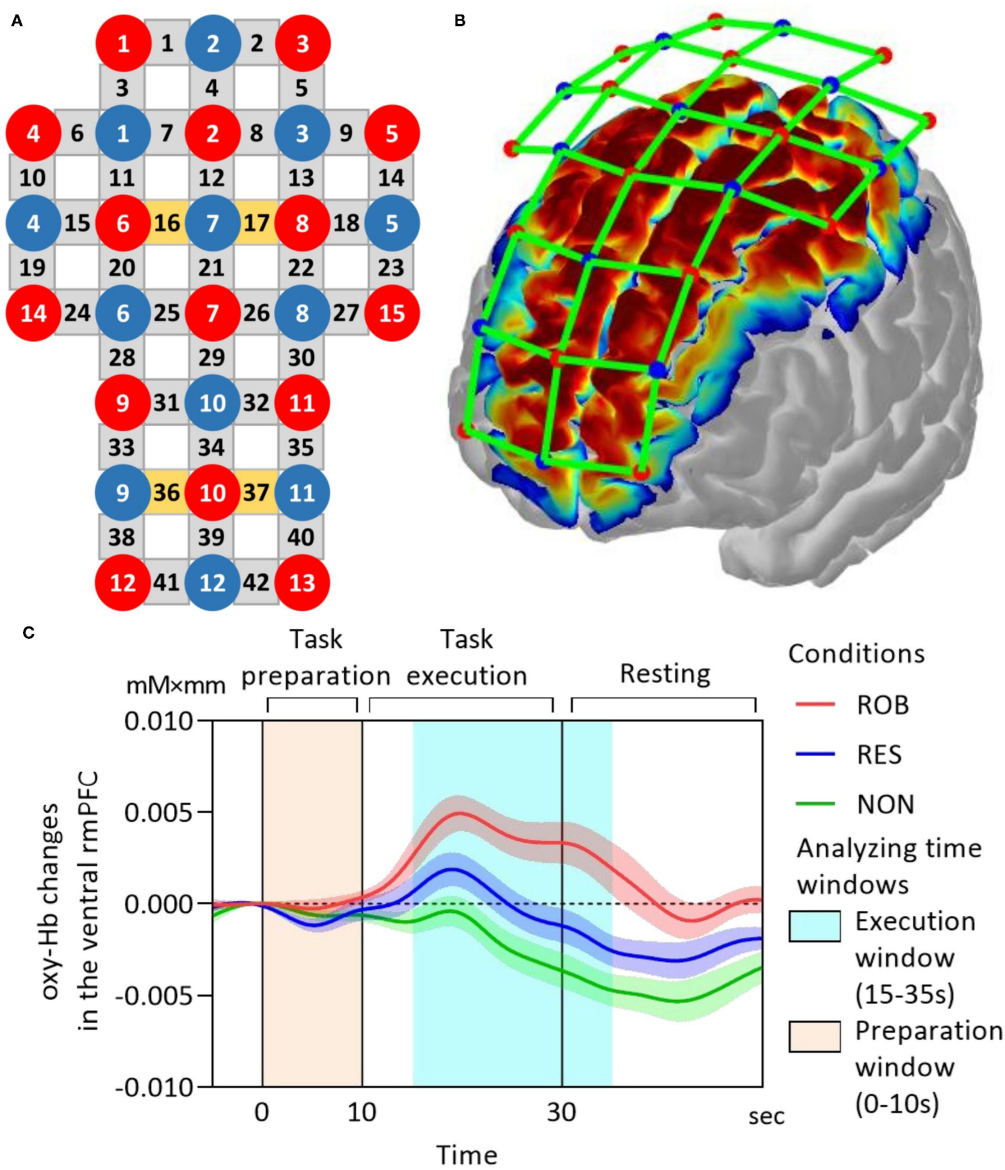
fNIRS data acquisition. **(A)** fNIRS optode layout design. Red and blue-filled circles represent light sources and detectors, respectively. Yellow and gray rectangles represent long separation channels with and without short separation channels, respectively. The midline central point (Cz) is located underneath the 12th channel. The source-detector distance is 3 cm. **(B)** The cortical mapping shows estimated spatial information of the measurement on the surface of the cerebral cortex using the current fNIRS optode configuration. **(C)** Grand-average oxy-Hb responses in the ventral rmPFC during ROB (red line), RES (blue line), and NON (green line). The task onset is at 0 on the x-axis. The time series were corrected to the baseline defined as the mean value over 2 s before the onset. fNIRS, functional near-infrared spectroscopy; oxy-Hb, oxy-hemoglobin; rmPFC, rostromedial prefrontal cortex. Data are expressed as the mean with standard error (SE).

Optodes were fixed to each participant's scalp using a customized head cap with a 30-probe layout (14 sources and 16 detectors) (Figure 2A). Optical probes comprised 42 long-separation channels (with a 3-cm source-detector distance), covering cerebral cortical regions including the rmPFC, dlPFC, premotor area (PM), primary motor cortex (M1), and primary somatosensory cortex (S1) (Figure 2B). As the fNIRS signal is derived from both regional cortical blood flow and scalp blood flow (Takahashi et al., 2011), we also used four short-separation channels (with a 1.5-cm source-detector distance) that measured hemodynamic signals from extracranial tissues (e.g., scalp and skull). Signals from these short channels were used for data processing. Probes were placed according to the international 10–20 electroencephalogram electrode system, with the midline central point of the scalp (Cz) positioned beneath the 12th channel. To obtain channel-related anatomical information, a 3D digitizer (FASTRAK, Polhemus, Colchester, Vermont, USA) was used to record the 3-dimensional position of each optical probe and four reference landmarks including nasion, Cz, left auricular, and right auricular points. Channel locations were estimated from coordinates of optodes and reference points using the Montreal Neurological Institute standard space coordinates. To anatomically label fNIRS channels, probabilistic mapping between each fNIRS channel and its corresponding Brodmann Areas (BA) was performed using the open-source software package Statistical Parametric Mapping for NIRS (NIRS-SPM, BISP Lab, Daejeon, Korea) implemented in MATLAB (MathWorks, Natick, Massachusetts, USA). The channel sets for regions of interest (ROIs) were selected based on BAs and anatomical locations of cortical areas for each participant (Table 1). To investigate neural signals in subregions of the rmPFC, channels within BA 10 were classified into two subdivisions along the ventral-dorsal axis: the ventral rmPFC, located in the ventral-middle part of BA 10 adjacent to BA 11 (orbital prefrontal cortex); and the dorsal rmPFC, positioned in the dorsal-middle part of BA 10 adjacent to BA 9 (dorsolateral prefrontal cortex).

**Table 1:** Anatomically labeled fNIRS channel locations using Brodmann areas.

**Channels**	**Brodmann areas (BA)**	**Regions of interest (ROI)**	**Abbreviation**
1, 3	BA 1, 2, 3	Right primary somatosensory cortex	Right S1
2, 5	BA 1, 2, 3	Left primary somatosensory cortex	Left S1
6, 7, 10	BA 4	Right primary motor cortex	Right M1
8, 9, 14	BA 4	Left primary motor cortex	Left M1
11, 15, 16, 19	BA 6	Right premotor and supplementary motor cortex	Right PM
13, 17, 18, 23	BA 6	Left premotor and supplementary motor cortex	Left PM
28, 31, 33	BA 9, 46	Right dorsolateral prefrontal cortex	Right dlPFC
30, 32, 35	BA 9, 46	Left dorsolateral prefrontal cortex	Left dlPFC
34, 36, 37	BA 10	Dorsal rostromedial prefrontal cortex	Dorsal rmPFC
39, 31, 32	BA 10	Ventral rostromedial prefrontal cortex	Ventral rmPFC

*The channel sets for ROIs were individually adjusted for each participant based on NIRS-SPM probabilistic mapping*.

*fNIRS, functional near-infrared spectroscopy; ROIs, regions of interest; NIRS-SPM, Statistical Parametric Mapping for NIRS; BA, Brodmann Areas; rmPFC, rostromedial prefrontal cortex; dlPFC, dorsolateral prefrontal cortex; PM, premotor area; M1, primary motor cortex; S1, primary somatosensory cortex*.

To deal with motion artifacts possibly induced by body movements, fNIRS signals were processed using a method based on moving SD and spline interpolation (Scholkmann et al., 2010). This approach computed the SD of each data segment and identified motion artifacts based on the SD threshold. The data segments containing the motion artifact would then be spline interpolated. A band-pass filter with a 0.01–0.1 Hz cutoff frequency range was then applied to remove concomitant systemic responses from the signal. Next, we employed a technique called direct subtraction to remove these extracerebral hemodynamic components from the neural data. Each long channel was linked with the short channel that is closest to it. The corrected hemodynamic response was obtained by subtracting the corresponding short channel signal from the long channel signal. The preceding data analyses were carried out with the use of commercial fNIRS analysis software (Advanced ROI; WAWON DIGITECH, Japan). The oxy-Hb time-series in each channel were corrected to baseline values, determined as the mean over 2 s prior to the onset of the task period. Subsequently, the oxy-Hb time-series were averaged across trials and ROI-wise channels to generate ROI time-series for each condition. Based on the ROI time-series, mean oxy-Hb changes were used as an index of cortical activation and were calculated separately for each task phase, namely the preparation phase (0 to 10 s) and execution phase (15 to 35 s), with the task onset set at 0 s. The time window for the execution phase was defined based on the temporal characteristics of blood oxygenation hemodynamic responses.

Based on previous evidence (Schroeter et al., 2003), we employed effect size as an index of brain activation due to its robustness to differential path-length factors. For each channel, the effect size in each execution period was calculated as the difference between the mean oxy-Hb changes in the execution window (15–35 s) and baseline window (−5–5 s), divided by the SDs of the baseline window. For statistical analysis, the effect sizes were averaged across trials and ROI-wise channels to generate the average effect sizes for each condition.

The fNIRS data were also analyzed using general linear model-based approaches in NIRS-SPM (Ye et al., 2009), in which actual hemodynamic responses were compared to models of theoretical responses to calculate *t*-statistics for each channel. Three comparisons were investigated: ROB vs. RES, ROB vs. NON, and RES vs. NON. The general linear model method compared the theoretical model with actual hemodynamic responses to calculate *t*-statistics for each channel. Group-level *t*-statistic maps were generated to visualize execution-related activation for each comparison at a *p* < 0.0167 or 0.05/3. The results on the map were interpolated across participants and between channels due to anatomical variation (e.g., head shape and size) and channel spacing, respectively (Ye et al., 2009). Alignment of analysis results based on mean oxy-Hb changes, average effect sizes of hemodynamic responses, and group-level *t*-statistic maps would help better confirm task-related cortical activity.

### Statistical Analysis

The size of the extracted features for hemodynamic responses are as follows: four consecutive trials in each three (ROB, RES, and NON) conditions, total 10 ROIs selected from 38 channels. One-way repeated-measures analysis of variance (ANOVA) tests were used to examine the effect of conditionon indices of cortical activation (mean oxy-Hb changes and average effect sizes of hemodynamic response) for each ROI. To examine the brain-behavior relationship, we conducted Pearson's correlation analysis between differences in execution-related mean oxy-Hb changes in rmPFC subregions and average SD values. Bonferroni *post-hoc* tests were applied for multiple comparisons (α = 0.0167). SPSS statistical package version 19.0 (IBM, Co. Ltd, New York, USA) was used for statistical analysis. *P* ≤ 0.05 were considered statistically significant.

## Results

### Behavioral Results

**Figure 3:**
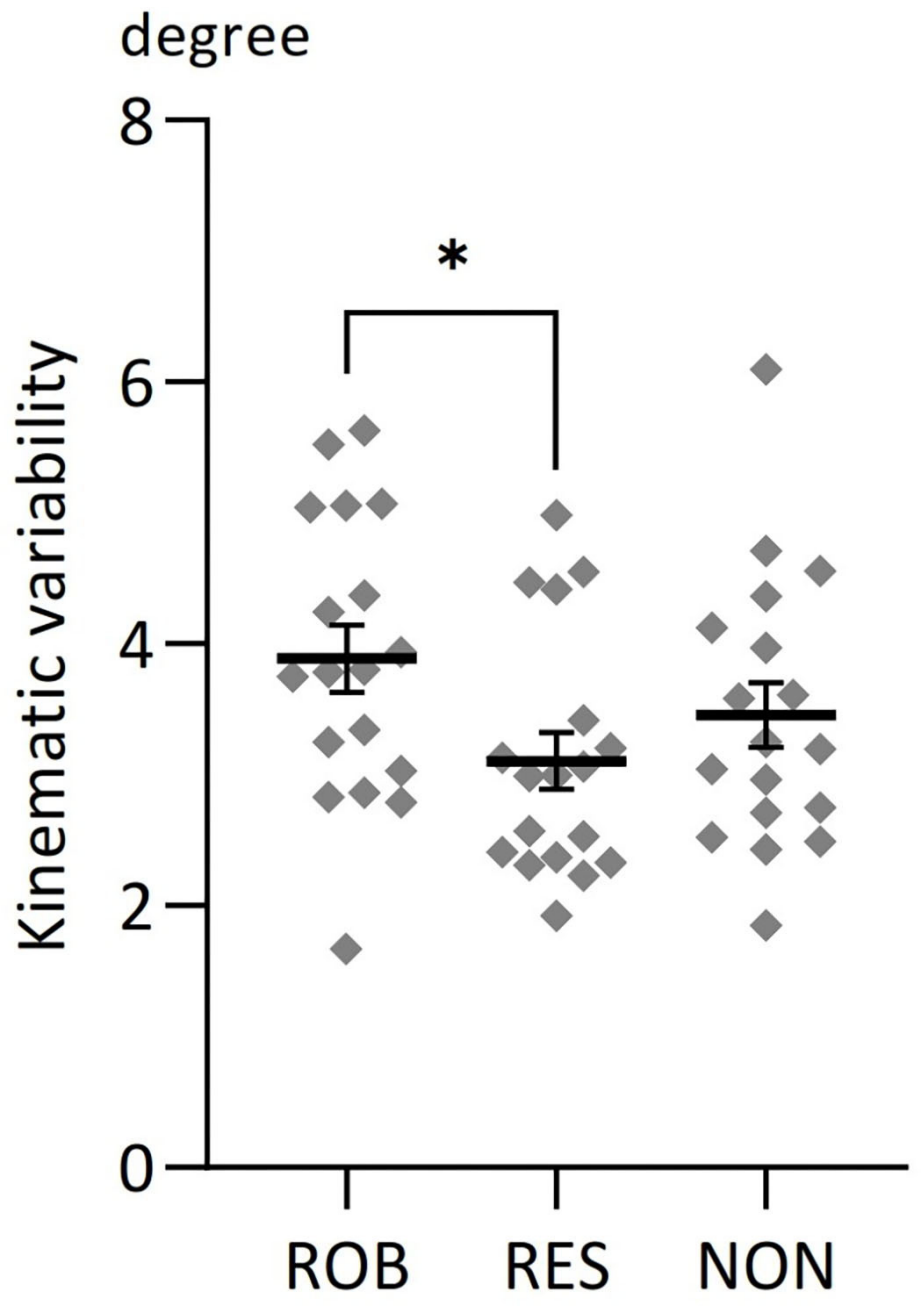
Average changes in kinematic variability for three conditions. Comparisons of average changes in kinematic variability for ROB, RES, and NON. *p < 0.05. Data are expressed as the mean with standard error (SE).

One-way repeated measures ANOVA revealed significant differences in average SD values among conditions [*F*_(2,34)_ = 3.364, *p* = 0.046, Figure 3]. *Post-hoc* testing using Bonferroni correction revealed that kinematic variability was significantly higher during ROB than during RES (*p* = 0.033).

### fNIRS Results

Illustrations of grand-average ROI time-series of mean oxy-Hb changes in the ventral rmPFC during ROB, RES, and NON are presented in Figure 2C.

**Figure 4:**
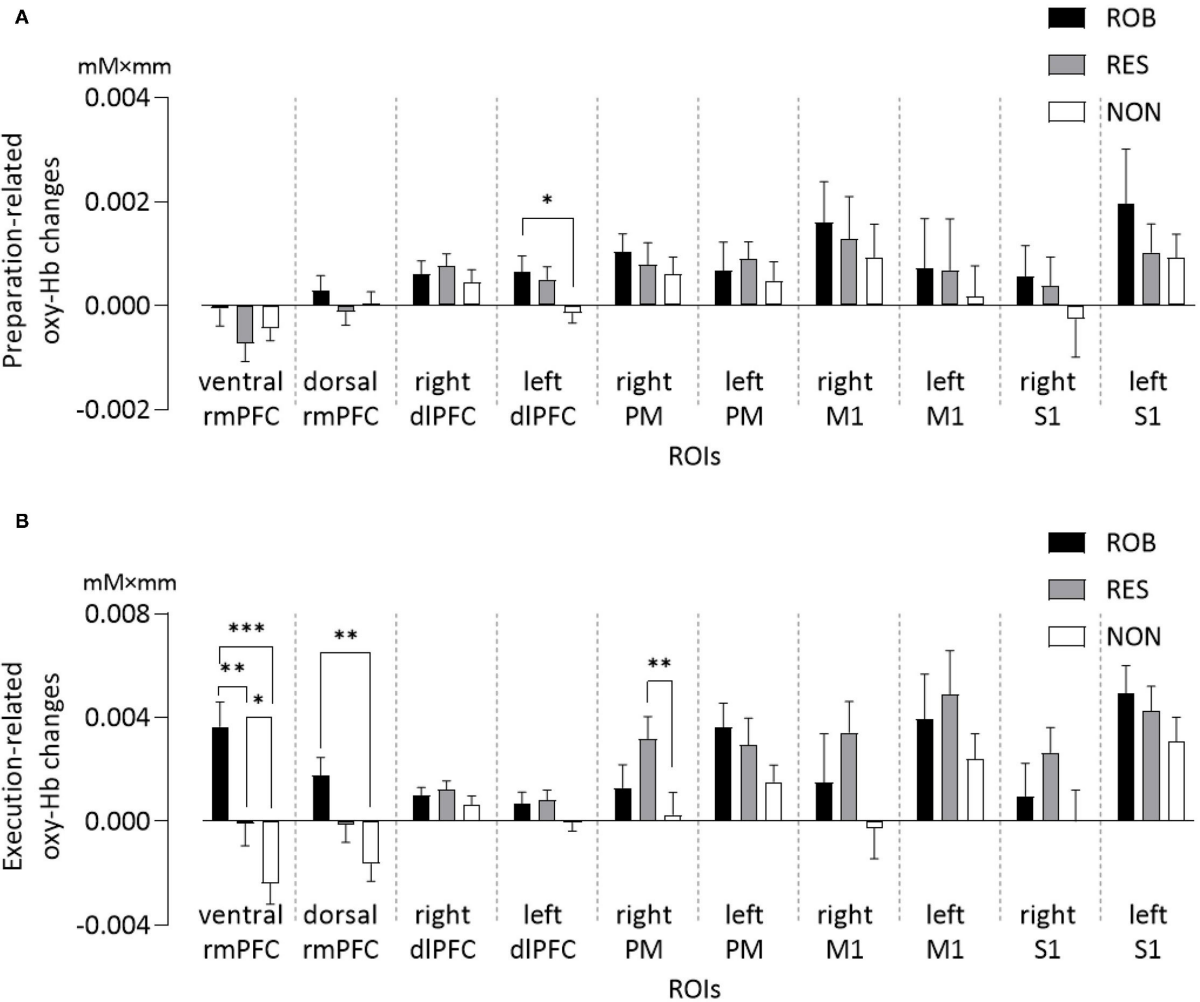
Average changes in oxy-Hb concentration of ROIs for three conditions. Comparisons of **(A)** preparation-related, and **(B)** execution-related oxy-Hb changes of ROIs for ROB, RES, and NON. oxy-Hb, oxy-hemoglobin; ROIs, regions of interest; rmPFC, rostromedial prefrontal cortex; dlPFC, dorsolateral prefrontal cortex; PM, premotor area; M1, primary motor cortex; S1, primary somatosensory cortex. *p < 0.05, **p < 0.01, ***p < 0.001. Data are expressed as the mean with standard error (SE).

One-way repeated measures ANOVA revealed significant differences in preparation-related mean oxy-Hb changes among conditions in the left dlPFC [*F*_(2,34)_ = 4.28, *p* = 0.022] (Figure 4A). Bonferroni *post-hoc* testing revealed that oxy-Hb responses in the left dlPFC were significantly higher during ROB and RES than during NON (*p* = 0.039 and *p* = 0.012, respectively).

One-way repeated measures ANOVA also revealed significant differences in mean oxy-Hb changes among conditions in the three ROIs (Figure 4B): ventral rmPFC [*F*_(2,34)_ = 22.10, *p* < 0.001], dorsal rmPFC [*F*_(2,34)_ = 11.26, *p* < 0.001], and right PM [*F*_(2,34)_ = 5.80, *p* = 0.007]. Oxy-Hb responses in the ventral rmPFC region were stronger during ROB than during RES (*p* = 0.002) and NON (*p* < 0.001). Cortical activation in the ventral rmPFC was significantly greater during RES than during NON (*p* = 0.020), whereas activity in the dorsal rmPFC was significantly greater during ROB than during NON (*p* = 0.001). Oxy-Hb responses in the right PM were significantly higher during RES than during NON (*p* = 0.005).

One-way repeated measures ANOVA revealed significant differences in average effect sizes of hemodynamic responses in the ventral rmPFC [ROB: 4.07 ± 1.11, RES: 0.28 ± 1.02, NON: −2.26 ± 0.81; *F*_(2,34)_ = 17.872, *p* < 0.001], dorsal rmPFC [ROB: 2.76 ± 0.68, RES: −1.18 ± 1.08, NON: −2.08 ± 0.87; *F*_(2,34)_ = 10.74, *p* < 0.001], and right PM [ROB: 0.83 ± 0.64, RES: 1.97 ± 0.43, NON: −0.46 ± 0.54; *F*_(2,34)_ = 6.942, *p* = 0.003]. Significantly greater effect sizes were observed in ROB than in RES (ventral rmPFC: *p* = 0.019, dorsal rmPFC: *p* = 0.011) and NON (ventral rmPFC: *p* < 0.001, dorsal rmPFC: *p* < 0.001) in ventral and dorsal rmPFC regions. Further, in the ventral rmPFC, a significantly greater effect size was observed in RES than in NON (*p* = 0.040), and in the right PM, the effect size was significantly greater during RES than during NON (*p* = 0.001). The results are detailed in the Supplementary Tables S1,S2.

**Figure 5:**
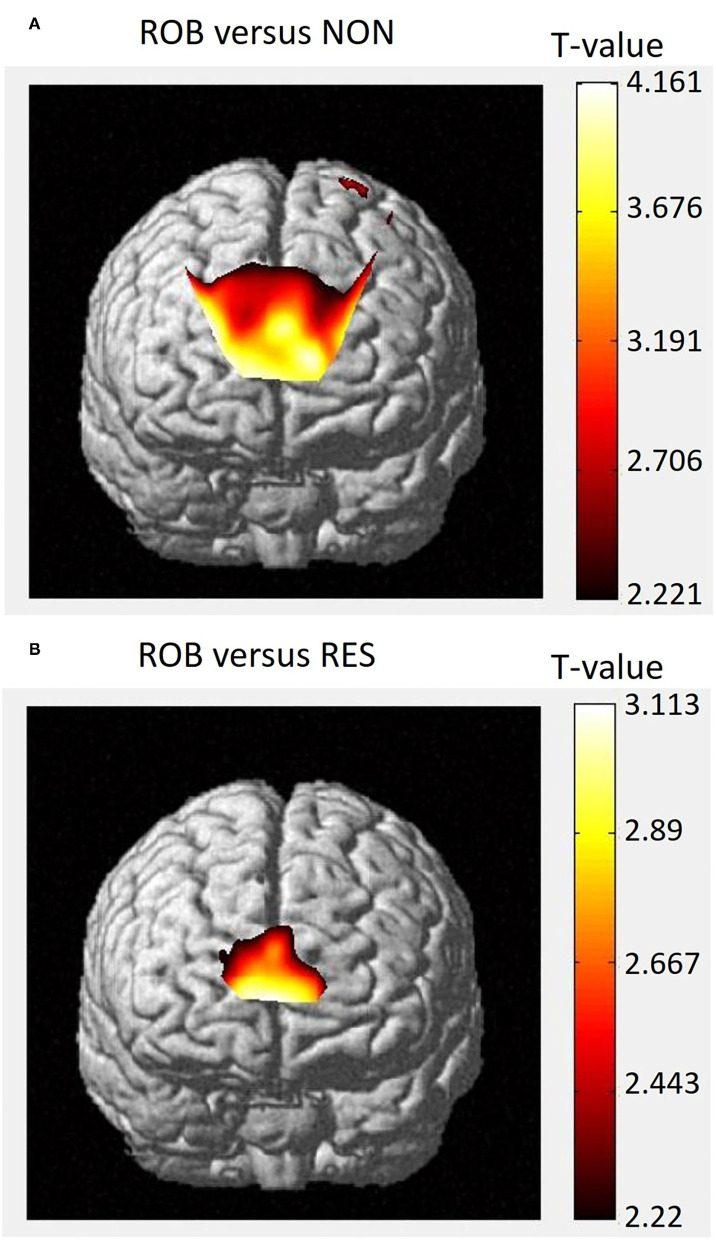
Group-level *t*-statistic map. Execution-related cortical activations, as determined by NIRS-SPM, for the comparisons between **(A)** ROB vs. NON, and **(B)** ROB vs. RES. Higher *t*-values represent relatively higher levels of cortical activation.

Group-level *t*-statistic maps are presented in Figure 5. Group analysis of the comparison between ROB and NON revealed significant activity in both the ventral and dorsal parts of the rmPFC (Figure 5A). For the comparison between ROB and RES, increased cortical activation was specifically identified in the ventral rmPFC (Figure 5B).

### Correlation Results

Correlation analyses of the relationship between differences in fNIRS data and task performance revealed that execution-related mean oxy-Hb changes in the dorsal and ventral rmPFC during ROB were positively correlated with average SD values (dorsal rmPFC: r = 0.473, *p* = 0.047; ventral rmPFC: r = 0.480, *p* = 0.044). However, neither of them survived after Bonferroni correction. Additionally, no significant association was observed between differences in cortical activity and behavioral measures, including VAS scores and the number of jerks in ROB.

## Discussion

The present study aimed to investigate the effects of a robot-assisted motor task on the cortical activity involved in the executive resource allocation using fNIRS in healthy participants. We hypothesized that human-robot interactions in ROB would activate rmPFC subregions to bias the allocation of processing resources toward external IP. Statistical analyses of hemodynamic changes and effect sizes of hemodynamic responses revealed greater activation in both the ventral and dorsal rmPFC during ROB than during NON. Furthermore, activity in the ventral rmPFC was greater during ROB than during RES. These findings aligned with the results of group-level NIRS-SPM t-statistic maps. These findings are novel, as the rmPFC activity associated with executive resource allocation during a robot-assisted motor task has not been previously investigated.

In accordance with our hypotheses, the comparison between ROB and NON revealed increased activation in the rmPFC, including the ventral and dorsal parts. Although aiming for the same movement goal, NON purely involves self-control movements associated with internal IP, whereas ROB requires user interaction with external robotic assistance, thus engaging high degrees of external IP. Moreover, our analysis showed no difference in sensorimotor cortical activation, including M1 and S1, between conditions, indicating that the contrast of ROB vs. NON is likely to reflect the different degrees of the allocation of processing resources toward external IP instead of motor control demand or sensory-perceptual load. Therefore, our findings resonate with previous evidence reporting similar brain patterns with increased rmPFC activation and indistinct sensorimotor activities during externally oriented tasks, relative to internally-oriented ones (Gilbert et al., 2006, 2007; Henseler et al., 2011). The engagement of the rmPFC in a high-order system that monitors behavioral demands and governs the allocation of processing resources toward external IP has been demonstrated (Gilbert et al., 2006; Burgess et al., 2007a; Simons et al., 2008; Henseler et al., 2011; Burgess and Wu, 2013). The rostral PFC, including the rostromedial region, is anatomically interconnected with supramodal cortices in the PFC, anterior temporal cortex, and cingulate cortex (Morán et al., 1987; Arikuni et al., 1994; Petrides and Pandya, 1999). These connections enable the rostral PFC to evaluate and modulate the multimodal integration of processed information from downstream sensory systems. Furthermore, the rmPFC exhibits coactivation with sensory association cortices, the orbitofrontal cortex, posterior cingulate cortex, striatum, and thalamus during externally engaged conditions (Henseler et al., 2011). These regions are known to form functional coordination that prioritizes the processing of external feedback (Mesulam et al., 2001; Kiehl et al., 2005; Sridharan et al., 2007; Williams et al., 2007; Diekhof et al., 2009). A prior fNIRS-based BCI study examined rmPFC hemodynamic responses during the Stroop task that is well-known to involve a high level of external IP (Schudlo and Chau, 2015). The results showed that signals in the rmPFC area may be used to accurately decode external or stimulus-oriented mental states, hence corroborating the view of the rmPFC's role in prioritizing external IP. Our results, together with prior findings, suggest that during human-robot interaction, the rmPFC activity is involved in top-down executive control that biases the allocation of processing resources toward external IP.

Notably, we found increased rmPFC activation in the ventral subregion, but not in the dorsal subregion, during ROB, compared to RES. Motor performances during both ROB and RES are likely to require participants to consider external influences by assistive and resistive torques, respectively. Compared to a relatively constant resistance, physical coordination with robotic assistance by assessing user intention appears to be more computationally demanding for somatosensory feedback processes, engaging higher degrees of external IP. Although our results found higher task performance with ROB than with RES, we found no association between differences in rmPFC activity and behavioral measures or subjective feedback of effort on task performance. The above findings suggest that the increased ventral rmPFC activation of ROB vs. RES is unlikely to represent task difficulty effect across conditions. Our results are supported by those of previous studies suggesting that the most anterior part of PFC, corresponding to the ventral rmPFC in this study, plays a critical role in biasing the allocation of processing resources toward the external IP according to current behavioral demands (Gilbert et al., 2006, 2007; Burgess et al., 2007b; Henseler et al., 2011; Burgess and Wu, 2013). A posterior-to-anterior organization in hierarchical control of cognition by the PFC has been proposed (Koechlin and Summerfield, 2007; Rahnev, 2017). Under this framework, progressively anterior subdivisions of the PFC operate as higher-order structures in the hierarchy, representing progressive information-processing aspects of cognition and deploying top-down control over more posteriorly located regions. Therefore, it is plausible to suggest that during a motor task with an assistive exoskeleton robot, the rmPFC, especially its ventral subregion as a core part, is crucial to the executive process that prioritizes the processing of somatosensory feedback induced by physical human-robot interactions.

Additionally, our analysis of preparatory activity across conditions found a modulated activation in the dlPFC of ROB vs. NON, whereas no difference in rmPFC was observed. Preparing for impending tasks engages internal or stimulus-independent cognition, whereby the brain exerts top-down control to proactively mobilize processing resources to ensure successful performance without external stimuli (Braver, 2012). This process has been linked to the neural pre-movement activity in the dlPFC (Miller and Cohen, 2001; Badre and Nee, 2018), consistent with our findings. In contrast, the executive resource allocation of the rmPFC operates based on an external or stimulus-oriented cognition (Burgess et al., 2007a), in which stimulation from distal sensory modalities is required (Dixon et al., 2014). The above findings indicate the functional dissociation of the rmPFC-centered executive process that drives the allocation of processing resources with the endogenous goal-directed preparation located on other prefrontal cortices. Therefore, our results further support the executive involvement of the rmPFC activity in prioritizing external IP during human-robot interactions.

Taken together, our results suggest the involvement of rmPFC in top-down executive control during the robot-assisted motor task. This high-level aspect of cognitive control may be essential for optimal human-robot interactions. Indeed, assistive exoskeleton robots deliver motion aid based on the user's motion intentions. Physical interaction between people and robots generates novel and extra sensory feedback that is experienced intuitively, which leads to high kinematic variability (Berger et al., 2019). This process, as demonstrated in this study, may lead to more processing of external information, requiring the top-down executive control of the rmPFC to externally bias the allocation of processing resources. Although the role of the rmPFC has not been directly investigated in previous studies of robot-assisted motor tasks, there is indirect evidence for the involvement of this region in human-robot interactions. Previous fNIRS research demonstrated that adaptation to robot-assisted motor tasks promotes the user's active engagement, which leads to greater variability associated with sensorimotor cortex activation (Berger et al., 2019). Based on recent fNIRS evidence on learning novel skillful movements, such a brain-behavior association may be governed by top-down modulation from the rmPFC (Ota et al., 2020; Kobayashi et al., 2021). Furthermore, previous neuroimaging research revealed that providing robotic assistance for voluntary limb movement may involve the fronto-centro-parietal network (García-Cossio et al., 2015; Youssofzadeh et al., 2016; Memar and Esfahani, 2018), which has been regarded as the top-down executive control network, of which the rmPFC area is a higher-order component (Peng et al., 2018b). Therefore, the current study, combined with earlier findings, suggests that top-down executive control involvement, particularly those relying on the rmPFC, is crucial to human-robot interactions during robot-assisted motor tasks.

### Implications

To the best of our knowledge, this study is the first one that investigates the role of rmPFC subregions in executive resource allocation during a robot-assisted motor task. Our findings contribute to a better understanding of an aspect of top-down executive control that determines and prioritizes the mode of information processing best suited to the current behavior. This executive process appears to be vital for adapting human-robot interactions, demanding further research into the role of the rmPFC in different robot-assisted modalities (e.g., assistive lower limb exoskeleton). As the use of assistive exoskeleton robots in life and rehabilitation grows more popular, gaining a deeper grasp of the neural mechanisms underlying human-robot interactions may have the potential to improve robotic technology for rehabilitation and ergonomics. Our results, as well as the analysis method and statistical methodology outlined here, provide a helpful baseline that future researchers can use to further examine human-robot interactions in healthy and clinical populations in more depth.

Furthermore, our results may strengthen the use of the fNIRS-based measurement of rmPFC activity as a useful brain indicator in rehabilitation. Due to its tolerance to motion artifacts and motor disabilities (Erdogan et al., 2019), hemodynamic responses of the rmPFC regions have been proposed as a potential signal source for BCI applications (Hong and Khan, 2017) and therapeutic interventions (Kohl et al., 2020; Ota et al., 2020). Top-down resource allocation toward external IP has been described as an external or stimulus-oriented state of cognition (Dixon et al., 2014). Our findings imply that such a cognitive state, which appears to be crucial for human-robot interactions, may be identified by rmPFC activity. This demonstrates the potential for rmPFC activity to aid in the functional outcome assessment of pre- and post-interventions with robot-assisted training. Future studies with longer task duration and follow-up assessment will help in further delineation of the role of the rmPFC in the performance of robot-assisted tasks.

The present study also contributes to further promoting the use of rmPFC signals in BCI applications. It has been demonstrated that the PFC regions contain sufficient information to accurately detect brain processes, including sensorimotor processes, cognitive functions, and mental states (Min et al., 2017; Khan et al., 2021; Liu et al., 2021). Our study has untangled one of the rmPFC's roles, which is related to stimulus-directed cognitive states. This feature of rmPFC activity may be important for effective human-machine communication where feedback from the computer to the user is also a key aspect. In future studies, attempts are encouraged to determine ways to combine inputs from the rmPFC and other regions to improve the interface between humans and machines.

The present study also highlights the capability of fNIRS to detect brain changes while participants performed robot-assisted motor tasks. This motivates the application of fNIRS in future studies on human-robot interactions and motion-related tasks in naturalistic settings.

### Limitations

This study has several limitations. First, the fNIRS analysis had low spatial resolution. Although this study focused on hemodynamic responses in large-scale cortical regions, it would be desirable for future studies to employ a highly dense fNIRS probe set to obtain more precise information about task-related neural signals. Second, our experimental tasks involved upper limb and body movements, which may have induced noise disturbance in the fNIRS data. To deal with motion artifacts, the participants' head movements were strictly suppressed across the three conditions using a chinrest. The extracerebral components were filtered out from the fNIRS data using signals from short-separation channels, and motion artifacts were corrected using a technique based on moving standard deviation and spline interpolation. Despite our best efforts, completely filtering noise from neural signals remains a challenge, necessitating further research with more suitable paradigms to overcome these motion artifacts. Third, our research solely assessed task-related brain activity through regional hemodynamic responses, which may not have taken into account the intricate relationships between cortical regions. The combination of functional connectivity among brain areas and regional cortical activities can help provide a complete understanding of the neural mechanisms underpinning human-robot interactions (Youssofzadeh et al., 2016). Future studies on the connectivity patterns of brain networks during robot-assisted tasks are required to expand on the current findings. Fourth, the setting parameters for HAL-SJ were standardized across all participants without taking individual kinematic characteristics into account. Future studies should consider personalized tuning for robotic assistance, which may help further investigate the relationship between brain activity and motor behavior.

## Conclusion

In conclusion, this fNIRS study assessed the cortical mechanisms of executive resource allocation during a robot-assisted motor task. Our study demonstrated that, as compared to manual motor tasks, robot-assisted task performance can increase brain activity in rmPFC subregions. This rmPFC activation may be associated with the top-down executive control that supports human-robot interactions by prioritizing the processing of external information. Our study contributes to the previously unexplored understanding of how top-down executive control operates and supports human-robot interactions. As the usage of assistive exoskeleton robots becomes more common in life and rehabilitation, this study may serve as a useful baseline for future researchers to investigate human-robot interactions in healthy and clinical populations in greater depth. Further investigations with follow-up assessment are warranted to extend the findings of our study.

## Data Availability Statement

The original contributions presented in the study are included in the article/Supplementary Materials, further inquiries can be directed to the corresponding author.

## Ethics Statement

The studies involving human participants were reviewed and approved by Human Ethics Committee of Hiroshima University. The patients/participants provided their written informed consent to participate in this study.

## Author Contributions

DL, KW, HO, NF, and SU performed experiments. KM, NI, ST, YI, and TI supported technically. TI, TO, HN, NF, and SU interpreted results of experiments. DL and SU prepared the figures and table. DL, NF, and SU drafted the manuscript. DL, KM, TI, HA, OA, TO, HN, NF, and SU approved the final version of manuscript. DL, HA, OA, TO, HN, NF, and SU edited and revised the manuscript. All authors contributed to the article and approved the submitted version.

## Funding

This research was supported by a Hiroshima ROBOCARE CENTER, Grant-in-Aid for Scientific Research from the Japanese Ministry of Education, Culture, Sports, Science, and Technology (17K01503 and 20H04044; SU), and research funds from Hiroshima University (187018).

## Conflict of Interest

The authors declare that the research was conducted in the absence of any commercial or financial relationships that could be construed as a potential conflict of interest.

## Publisher's Note

All claims expressed in this article are solely those of the authors and do not necessarily represent those of their affiliated organizations, or those of the publisher, the editors and the reviewers. Any product that may be evaluated in this article, or claim that may be made by its manufacturer, is not guaranteed or endorsed by the publisher.
