# Supplementary material for: Involvement of the Rostromedial Prefrontal Cortex in Human-Robot Interaction: fNIRS Evidence From a Robot-Assisted Motor Task
Source: Front Neurorobot. 2022 Mar 17;16:795079. doi: 10.3389/fnbot.2022.795079 (PMC8970051; doi:10.3389/fnbot.2022.795079)
Supplement: Supplementary file 1 [file Data_Sheet_1.pdf]

Supplementary Table S1: Results of one-way repeated-measures analysis of variance (ANOVA) on average effect sizes of hemodynamic responses. ROI, region of interest; SD, standard deviation; F, F-measure; rmPFC, rostromedial prefrontal cortex; dlPFC, dorsolateral prefrontal cortex; PM, premotor area; M1, primary motor cortex; S1, primary somatosensory cortex.

**ROI**	**ROB**		**RES**		**CON**		**One-way ANOVA**	
	**Mean**	**SD**	**Mean**	**SD**	**Mean**	**SD**	**F**	***p*-value**
Right S1	0.202	0.495	1.236	0.446	-0.089	0.401	3.062	0.060
Left S1	2.367	0.463	2.064	0.753	1.519	0.442	0.678	0.514
Right M1	0.174	0.733	0.760	0.477	-0.493	0.561	1.501	0.237
Left M1	1.504	0.431	1.731	0.488	0.915	0.343	1.260	0.296
Right PM	0.833	0.635	1.968	0.425	-0.458	0.537	6.942	0.003
Left PM	2.604	0.705	2.136	1.016	0.616	0.545	2.399	0.106
Right dlPFC	0.211	0.839	0.206	1.051	-1.120	-0.620	1.044	0.363
Left dlPFC	0.033	1.139	0.656	1.099	-1.227	0.532	1.886	0.167
Dorsal rmPFC	2.757	0.675	-1.184	1.084	-2.082	0.874	10.744	<0.001
Ventral rmPFC	4.067	1.111	0.285	1.018	-2.262	0.808	17.872	<0.001

Supplementary Table S2: Results of the Bonferroni-corrected *post-hoc* comparisons after one-way repeated-measures ANOVAs on average effect sizes of hemodynamic responses. ROI, region of interest; SD, standard deviation; rmPFC, rostromedial prefrontal cortex; PM, premotor area.

**ROI**	**Contrast**	**Mean difference**	**SD**	***p*-value**
Right PM	RES – CON	2.427	0.565	0.001
Dorsal rmPFC	ROB – RES	3.941	1.164	0.011
	ROB – CON	4.840	0.929	<0.001
Ventral rmPFC	ROB – RES	3.782	1.213	0.019
	ROB – CON	6.329	1.042	<0.001
	RES – CON	2.546	0.920	0.040
